# PROFICS: A bacterial selection system for directed evolution of proteases

**DOI:** 10.1016/j.jbc.2021.101095

**Published:** 2021-08-19

**Authors:** Christina Kröß, Petra Engele, Bernhard Sprenger, Andreas Fischer, Nico Lingg, Magdalena Baier, Christoph Öhlknecht, Bettina Lier, Chris Oostenbrink, Monika Cserjan-Puschmann, Gerald Striedner, Alois Jungbauer, Rainer Schneider

**Affiliations:** 1 acib GmbH, Graz, Austria; 2 Institute of Biochemistry and Center for Molecular Biosciences Innsbruck, University of Innsbruck, Innsbruck, Austria; 3 Department of Biotechnology, University of Natural Resources and Life Sciences, Vienna, Austria; 4 Institute of Molecular Modeling and Simulation, University of Natural Resources and Life Sciences, Vienna, Austria

**Keywords:** biotechnology, enzyme mutation, circular permutation, caspase, *E. coli*, directed evolution, tag cleavage, viral protease, 6H-tag, hexa histidine tag, ATCase, aspartate transcarbamoylase, *E. coli*, *Escherichia coli*, ep PCR, error-prone polymerase chain reaction, IPTG, isopropyl β-d-1-thiogalactopyranoside, MAP, methionine aminopeptidase, oe PCR, overlap extension polymerase chain reaction, PROFICS, protease optimization *via* fusion-inhibited carbamoyltransferase-based selection

## Abstract

Proteases serve as important tools in biotechnology and as valuable drugs or drug targets. Efficient protein engineering methods to study and modulate protease properties are thus of great interest for a plethora of applications. We established PROFICS (PRotease Optimization *via* Fusion-Inhibited Carbamoyltransferase-based Selection), a bacterial selection system, which enables the optimization of proteases for biotechnology, therapeutics or diagnosis in a simple overnight process. During the PROFICS process, proteases are selected for their ability to specifically cut a tag from a reporter enzyme and leave a native N-terminus. Precise and efficient cleavage after the recognition sequence reverses the phenotype of an *Escherichia coli* knockout strain deficient in an essential enzyme of pyrimidine synthesis. A toolbox was generated to select for proteases with different preferences for P1′ residues (the residue immediately following the cleavage site). The functionality of PROFICS is demonstrated with viral proteases and human caspase-2. PROFICS improved caspase-2 activity up to 25-fold after only one round of mutation and selection. Additionally, we found a significantly improved tolerance for all P1′ residues caused by a mutation in a substrate interaction site. We showed that this improved activity enables cells containing the new variant to outgrow cells containing all other mutants, facilitating its straightforward selection. Apart from optimizing enzymatic activity and P1′ tolerance, PROFICS can be used to reprogram specificities, erase off-target activity, optimize expression *via* tags/codon usage, or even to screen for potential drug-resistance-conferring mutations in therapeutic targets such as viral proteases in an unbiased manner.

Proteases are important enzymes in various industry sectors and are used for a wide range of applications. Wild-type proteases have evolved to cut a specific group of proteins, while for therapeutic, diagnostic, and biotechnological applications, a more diverse substrate specificity is needed (1). They often lack the necessary stability (2, 3), efficiency (4), specificity (5), or are not functional under the desired reaction conditions. Other industrially important characteristics of proteases are the incidence of their recognition site and their ability to create an authentic N-terminus (for a review see (6)). A toolbox with a set of similar proteases fulfilling these criteria and optimized for specific conditions and substrates would allow efficient processing of a wide range of substrates.

In order to turn wild-type proteases into optimized, therapeutically, diagnostically, and industrially applicable enzymes, we developed a versatile selection system, which can be applied to many different proteases and demonstrated its functionality with two viral proteases (pestiviral N-terminal autoprotease (N^pro^) (7) and Severe Acute Respiratory Syndrome Coronavirus-2 (SARS-CoV-2) main protease (M^pro^) (8)) and a human caspase that was optimized for tag cleavage (9). The two viral proteases process the precursor polyprotein of their respective virus at specific sites to release mature proteins. N^pro^ is able to release itself N-terminal of all amino acid residues except proline (10), whereas M^pro^ has a high specificity for a tetrapeptidic recognition site (11). First experiments for the selection system were executed with the autoprotease N^pro^ because it is a well-studied, robust enzyme (7) that acts cotranslationally (12) and is already used in industrial processes (10, 13, 14). In later experiments, we used M^pro^, the main protease of the current pandemic causing SARS-CoV-2, which is one of the prime targets for corona virus disease (COVID-19) drug design (8, 11, 15, 16).

With adaptions, caspases can be ideal enzymes for the cleavage of fusion tags. As key enzymes in apoptosis and inflammatory processes, caspases have a very high specificity and high efficiency when cleaving their natural substrates. However, they have hardly been considered for tag cleavage due to some unfavorable characteristics (17, 18). Their activation is tightly controlled, they are expressed as inactive zymogens and have to undergo cleavage and form dimers of heterodimers to become fully functional (19), which complicates their production and hampers their biotechnological application. While caspases principally tolerate most amino acids C-terminal of the cleavage site (the so-called prime site (20)) and are able to create native N-termini, the prime site residues hugely influence their activity (21). The interference is in particular caused by the first amino acid after the cleavage site (P1′ residue) (22).

Especially caspase-2 is an ideal candidate for proteolytic cleavage at industrial scale due to its extended recognition site (termed the unprimed site; adjacent N-terminal to the site of cleavage) (23). We have established a constitutively active, circularly permuted caspase-2 (cp caspase-2), which not only has a very high specificity but can also be easily and in high quantities produced in *Escherichia coli*, thus overcoming a main obstacle in the industrial usability of caspases (9). The circular permutation increases the enzyme’s activity compared with the wild-type, but the prime site residue still has a great influence on cleavage kinetics.

Here we show that these last drawbacks of cp caspase-2 can be resolved with PROFICS (PRotease Optimization *via* Fusion-Inhibited Carbamoyltransferase-based Selection), a method for directed evolution. It enables the optimization of this and other proteases by random mutation and the selection for variants improved in terms of activity and P1′ tolerance or with altered specificity.

### Directed evolution, screening, and selection

Directed evolution is the iterative process of gene mutation and subsequent screening or selection for improved properties (for a review see (24)). After each step, the most promising candidates are used as templates for a new round of mutation and screening or selection. This strategy can be repeated until the desired features are obtained.

Mutations can be introduced in the gene either randomly or by rational design (24). Random mutation can create almost countless variants. If, for example, only six positions in a protein are permuted to all canonical amino acids, their combinations form a library of 64 million candidates. Screening all those variants can be very challenging. It also shows that a crucial step in any *in vivo* system is the transformation efficiency of the host cell, which defines the size of the gene pool.

The number of variants can be decreased by targeted approaches, which use structural data to find the protein domains responsible for suboptimal characteristics and to define beneficial mutations (25). Öhlknecht *et al.* (26) have used an *in silico* approach to optimize cp caspase-2. Mutations that improve the P1′ tolerance were predicted by structural comparison of different caspases and confirmed in *in vitro* experiments.

The second step of directed evolution is finding improved variants among the abundance of mutants. For that purpose, an effective high-throughput screening or a selection system with a specific readout is needed. In screening methods, every single variant of the library must be tested, whereas during selection, proteins with undesired properties are eliminated and functional mutants separated.

### Circular permutation of aspartate transcarbamoylase

Circular permutation of a protein combines the circularization of a polypeptide backbone by covalent linkage of the N- and C-termini and opening of the chain at a different site. The order of the amino acids and chain connectivity are changed, but in most cases the tertiary structure of the protein remains almost unaltered (27). It has been shown that chain connectivity is not needed for proper folding and protein activity (28) and that allosteric properties are not influenced by circular permutation (29).

Aspartate transcarbamoylase (also aspartate carbamoyltransferase, ATCase, EC 2.1.3.2) is an essential *E. coli* enzyme and the catalyst in the committed step of the pyrimidine *de novo* biosynthesis, the end product of which is cytidine triphosphate (CTP). In the *E. coli* genome, all ATCase subunits are encoded on the same operon, the *pyrBI* operon (30).

ATCase has a molecular size of 310 kDa, comprises regulatory (R, r-chain) and catalytic (C, c-chain) polypeptide chains, and is highly organized. The dodecameric holoenzyme C6R6 consists of three regulatory dimers, 17 kDa each, and two catalytic trimers, 34 kDa each. The active sites are located at the interface of the catalytic subunits (31). If separated, the catalytic and regulatory chains reassemble into an active dodecameric holoenzyme with the same allosteric properties as the native enzyme (32).

It has been shown that circularly permuted catalytic chains of ATCase, constructed by covalent linkage of residues Arg^306^ and Ala^1^ of the wild-type c-chain and introduction of new termini at certain sites, can form active and stable ATCase-like molecules in combination with r-chains (33). The c-chain tolerates the opening of the peptide backbone at several positions without losing its ability to form catalytic trimers (29, 34). These findings by Zhang and Schachman prompted us to establish a novel protease selection system based on circularly permuted (cp) ATCase as an essential selectable marker.

## Results

The concept of the herein described protease selection system PROFICS is based on the idea that if the new termini of a circularly permuted enzyme are in a region with severe steric constraints, addition of amino acids to either terminus could inhibit correct folding.

**Figure 1:**
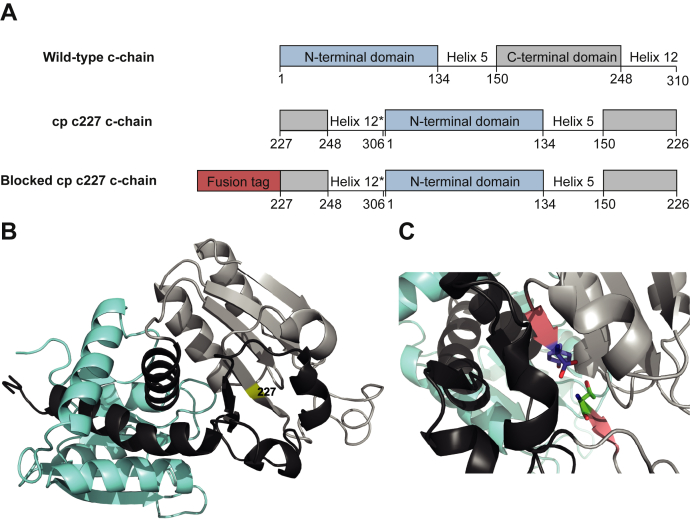
**Structural representation of *E. coli* ATCase catalytic chain.***A*, schematic amino acid sequences of ATCase wild-type c-chain, cp c227 c-chain, and N-terminally blocked cp c-chain by fusion of a peptide or protein. Circular permutation was realized by truncating the last four residues from helix 12 (marked with ∗) and connection of residues 306 and 1. The new N-terminus starts with Met^227^ and residue Tyr^226^ forms the new C-terminus. *Numbers* indicate residues in wild-type c-chain. Edited from (29). *B*, structural overview of ATCase c-chain (PDB ID 6AT1 (54)) with N-terminal (*blue*) and C-terminal domain (*grey*) connected by the two main helices 5 and 12 (*black*). The starting methionine of cp ATCase (*yellow*) at position 227 is located inside a β-sheet in the compact core structure of the c-chain. *C*, detailed visualization of the position for circular permutation. Spatial constraints within the β-sheet in c-chains (*red*) containing the new termini (N-terminus Thr^228^, *green*, C-terminus Tyr^226^, *blue*) of cp ATCase with starting methionine Met^227^ deleted.

We identified the active circular permutant cp ATCase c227 established by Zhang and Schachman (29) (in the interest of readability from now on cp ATCase) as suitable reporter enzyme with the desired prerequisites to establish a protease selection system. The new N-terminus starts with residue Met^227^ (Fig. 1, *A* and *B*, number of amino acids in the wild-type c-chain). Stable and active enzymes form when this cp c-chain is expressed together with wild-type r-chains. The new N- and C-termini of its cp c-chain are located in the C-terminal domain, inside a core structure of the protein (29) with little spatial flexibility (Fig. 1*C*). Hence, we expected that the addition of proteins or peptides to either of the termini would disturb the formation of active enzyme and block its catalytic activity. Specific proteolytic removal of the attached sequence should recover function.

For our selection system, we thus created pyrimidine auxotroph cells containing cp ATCase variants inactivated by a respective fusion peptide or protein. When the blocking sequence is removed, the subunits spontaneously fold into active enzymes. Therefore, the cells can grow in pyrimidine-free media if supplemented with a protease suitable to restore the function of cp ATCase by specific cleavage. The protease abundance, activity, specificity, and P1′ tolerance define the survival of the cells. This direct approach and simple readout allow only proteases with the desired features to overcome the selective pressure. In an overnight process, the best candidates are selected from the abundance of variants. Compared with rational design, our method is much faster and enables finding unconventional and new solutions.

### PROFICS: Proof of concept using the autoprotease N^pro^

An *E. coli* BL21(DE3) knockout strain (*E. coli pyr*−) was created by deletion of the *pyrBI* operon and its replacement with a kanamycin resistance cassette. The strain is auxotroph for pyrimidines and can only survive in media containing pyrimidines or when the cells are supplemented with an expression vector comprising the genes for catalytic and regulatory ATCase subunits. Small modifications enable the adaption of the system for the selection of different proteases.

### N^pro^ autoprotease

**Figure 2:**
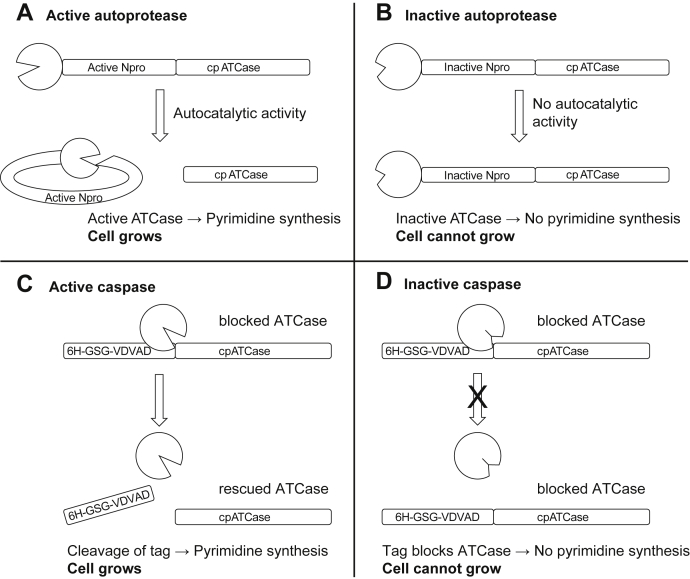
**Scheme of a selection system for proteases coexpressed with fused cp ATCase in *E. coli pyr***− **cells in pyrimidine-free medium.***A*, the active autoprotease N^pro^ is able to cleave itself autocatalytically from cp ATCase, the free cp ATCase is active and enables pyrimidine synthesis and cell growth. *B*, inactive N^pro^ is not able to cleave the cp ATCase fusion protein, cp ATCase cannot fold correctly and catalyze pyrimidine synthesis. Without pyrimidines the cell cannot grow. *C*, active caspase cleaves off the caspase-2 recognition tag (6H-GSG-VDVAD) from the cp ATCase and the knockout cell can produce pyrimidines to grow. *D*, inactive caspase does not cleave off the tag and the cp ATCase is blocked, the cell cannot grow under selection conditions.

To test the concept of PROFICS, the catalytic subunit of cp ATCase was N-terminally blocked by the fusion of either an active (Fig. 2*A*) or an inactive (Fig. 2*B*) variant of the viral autoprotease N^pro^. N^pro^ was inserted at Met^227^ of cp ATCase, inside a core structure, and prevented correct folding of the ATCase. The autoproteolytic cleavage by active N^pro^ during translation (12) restored the function of ATCase. Cells containing the unblocked cp ATCase or the active protease fusion protein grew in medium devoid of pyrimidines, whereas cells expressing inactive autoprotease did not survive the selective pressure. Formation of cleavage product was confirmed by a Western blot (experimental data in Fig. S1).

N^pro^ releases itself from the protein chain cotranslationally (35), which allows the ATCase to fold nearly undisturbed after removal of the autoprotease. After establishing that cp ATCase can be inactivated by the addition of N^pro^ to its N-terminus, we wanted to expand the application of the selection system from autoproteases with *cis* cleavage activity to endopeptidases with *trans* proteolytic activity.

### PROFICS applied to an endopeptidase

We chose a constitutively active, circular permutant of caspase-2 (cp caspase-2 (9)) as suitable endopeptidase. For its selection a hexa histidine-tag (6H-tag), followed by a short linker, and a caspase recognition site were added to the N-terminus of cp c-chains. We expected that the tag consisting of 14 amino acids would inactivate cp ATCase and cells expressing the tagged enzyme would only be able to produce pyrimidines when supplemented with a construct encoding a protease suitable to cleave the tag and restore the function of cp ATCase.

A scheme of PROFICS by coexpression of the two proteins is shown in Figure 2, *C* and *D*. To ensure that the cells contain both constructs, pETDuet-1 (*pyrB* and *pyrI*, ampicillin resistance) and pACYCDuet-1 (caspase, chloramphenicol resistance) vectors were used. They are designed for coexpression and differ in their origins of replication and antibiotic resistances.

The functionality of PROFICS for endopeptidases was tested in *E. coli pyr*− cells by coexpressing different cp ATCase constructs with the cp caspase-2 construct (Fig. S2). Cells expressing untagged cp ATCase formed colonies on selective plates within 24 h (Table 1). Cells that contained cp ATCase tagged with the caspase-2 recognition site VDVAD also formed colonies when complemented with the cp caspase-2 construct. When the tagged cp ATCase contained an altered recognition site (WEHD), which is not recognized and cleaved by cp caspase-2, no active cp ATCase formed and the cells were unable to grow on selective plates.

**Table 1:** List of plasmids used for double transformations (pACYC vector with/without protease, pETDuet vector with cp ATCase) into *E. colipyr*− cells and ability to overcome pyrimidine auxotrophy

Substrate plasmid MCSI/MCSII of pETDuet-1	Protease plasmid	
	Empty pACYC (Mock)	pACYC cp caspase-2
pyrI/cp pyrB	a	a
pyrI/6H-VDVAD-cp pyrB	b	a
pyrI/6H-WEHD-cp pyrB	b	b

a Cells able to form distinct colonies on selective agar within 24 to 48 h.

b Cells not able to form colonies on selective agar within 24 to 48 h.

### Selection efficiency

Mutation of a gene can create many inactive forms. The sensitivity of a selection system defines among how many inactive variants a single positive can be detected.

Several ratios (1:1–1:20,000 with constant volume and DNA concentration) of cp caspase-2 in pACYC plasmid and empty vector as mock were transformed into *E. coli pyr*− cells containing a cp ATCase construct with a 6H-VDVAD tag. Within 24 h distinct colonies formed. Sequencing analysis showed that all colonies contained the caspase construct, while cells containing the mock were unable to grow. The selection efficiency of our system on agar plates was 1:40,000, tested ratios were restricted by the transformation efficiency of the cells, which was 4 × 10^7^ cfu (colony forming units) per μg plasmid. Only 1 ng of plasmid was used for each transformation (40 μl total reaction volume) as transformations with low DNA concentration have a direct correlation between DNA amount and transformants, while higher amounts show a saturation (36).

### Construction of caspase mutant library

The number of mutants in the gene library and the transformation efficiency are crucial as they determine the probability to find optimized variants. Therefore, great care was taken in the creation of the library and the optimization of the steps described in the following.

Mutant gene libraries of cp caspase-2 were generated by error prone (ep) PCR and overlap extension (oe) PCR. The applied conditions caused in average three amino acid exchanges per caspase. The transformation efficiency is highly influenced by the quality of the ligation. Best results were achieved by ligating purified oe PCR product overnight.

To avoid inefficient double transformation of caspase libraries together with cp ATCase plasmids, electro competent *pyr*− cells containing the substrate plasmid were prepared. For transformations of the purified ligations into these cells, efficiencies up to 1.4 × 10^5^ cfu per preparation (8 ng ligated plasmid) were achieved, representing the possible pool of caspases for selection in a single experiment with our system. Ligations usually contain unligated or nicked plasmids, which reduces the transformation efficiency compared with plasmids amplified in cells.

### Novel caspase variant S9 identified *via* PROFICS

*E. coli pyr*− cells containing 6H-VDVAD-cp ATCase were transformed with a mutant library of cp caspase-2 and cultivated in selective media with an induction strength of 0.025 mM IPTG. The preparation contained about 5500 variants with 1 to 6 mutations per caspase gene. Selection was executed both with the flask and the agar plate assay. After 24 h colonies formed on plates. The liquid cultures turned turbid after 24 to 48 h and were then streaked on selective agar plates to obtain single colonies.

One caspase mutant was enriched in liquid culture and was also found in the plate assay (details in Table S1). This variant, termed S9, has two mutations: one silent mutation and Glu^105^ is exchanged to a valine (cp caspase-2 internal numbering, sequence in Supporting information). As the enrichment hinted toward beneficial properties, the variant was further characterized.

### Characteristics of S9 caspase variant

FRET assays showed that the cleavage activity (*k*_*cat*_/*K*_*M*_) of the S9 cp caspase-2 variant was significantly improved compared with cp caspase-2 (9). The increased activity is mainly due to an increase of the turnover number *k*_*cat*_, whereas the reaction rate *K*_*M*_ remained comparable (Table S2).

**Figure 3:**
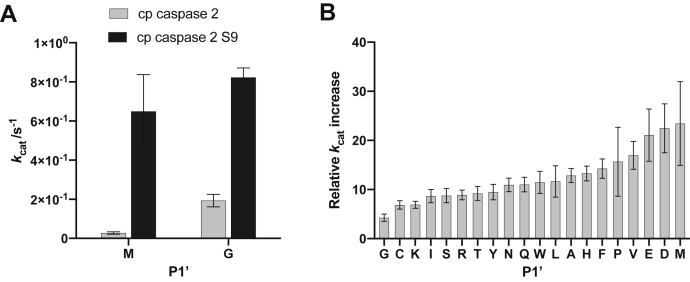
**P1′ tolerance of cp caspase-2 variant S9 compared with cp caspase-2.** Enzyme kinetics were measured with a peptide substrate-based FRET assay (n = 15, confidence interval 95%). *A*, comparison of cleavage activity of the two variants for substrates with amino acids methionine and glycine in the P1′ position. *B*, increase of turnover number *k*_cat_ of the cp caspase-2 mutant S9 compared with cp caspase-2 (data from (9)) for substrates with all 20 amino acids in P1′ position. In the interest of readability, single letter code was used for amino acids.

Strikingly, the *k*_*cat*_ of the new variant for the substrate with the amino acid methionine in the P1′ position was increased nearly 25-fold (Fig. 3*A*), which is the highest improvement among all tested substrates. Met was used as P1′ residue during the selection. The tolerance for all other P1′ residues was also increased (Fig. 3*B*). In addition to Met, the highest improvements were found for the least tolerated P1′ residues with low *k*_*cat*_/*K*_*M*_ values. Turnover numbers for substrates with Pro, Asp, Val, and Glu in the P1′ position were about 15 to 20 times higher compared with the unmutated variant after just one round of directed evolution.

### Influence of Glu^105^Val mutation

Molecular dynamics (MD) simulations explained the increased tolerance of variant S9, which includes the Glu^105^Val mutation, for all P1′ substrates. Structural visualization and hydrogen-bonding studies were conducted to evaluate the effects of the mutation on the altered promiscuity toward the P1′ substrates. The influence was analyzed using data from the crystallographic structure of caspase-2, PDB ID 1PYO (37), and trajectories from the MD simulations of both structures, with glutamate (caspase-2) and valine (S9 variant) at position 105.

As the substrate was modeled in the tetrahedral intermediate state, with a covalent bond to the catalytic cysteine, the classical MD simulations are representative of the catalytic step of the enzyme, and hence a comparison to the changes in *k*_*cat*_ rather than *K*_*M*_ is appropriate.

**Figure 4:**
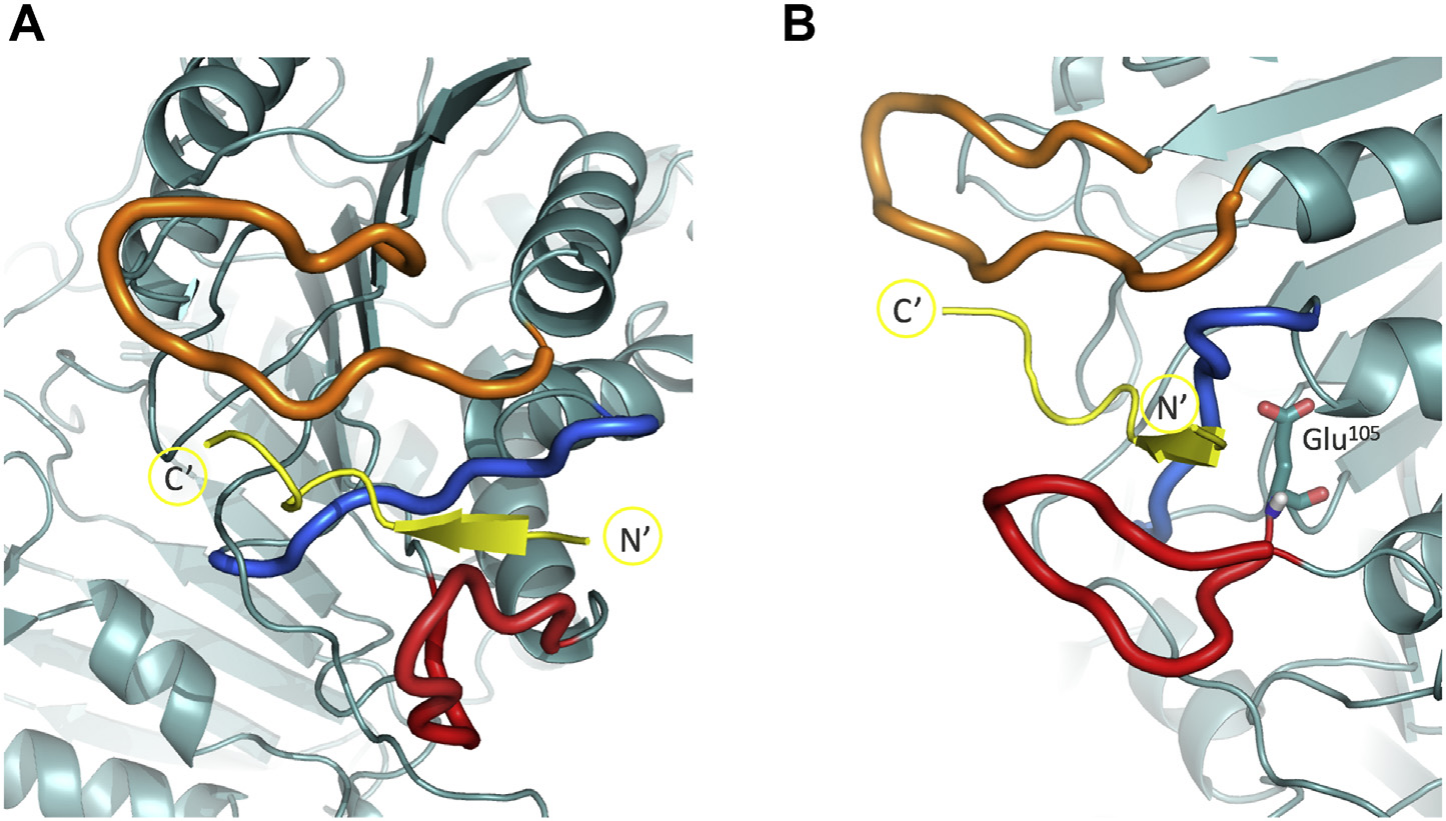
**Structural representation of caspase-2 active site with ligand bound (*yellow*).***A*, *upper loop* represented in *orange*, *lower loop* in *red*, *middle loop* in *blue*. *B*, view on the residue Glu^105^ that is located in close proximity to the *lower loop* (*red*) and has interaction sites with the *middle loop* (*blue*).

Caspase-2 interacts with its ligand primarily with three protein loops, here defined as upper, middle, and lower loop, as shown in Figure 4*A*. The upper loop (Gly^171^–Gly^180^, cp caspase-2 internal numbering) interacts with the primed site of the ligand C-terminal of the cleavage site (residues P1′–P4′), whereas the lower loop (Gly^93^–Glu^105^) interacts with the ligand’s unprimed site N-terminal of the cleavage site (residues P5–P1). The middle loop (Gly^47^–Arg^56^) forms important binding pockets and interacts with all ligand residues P5–P4′.

**Figure 5:**
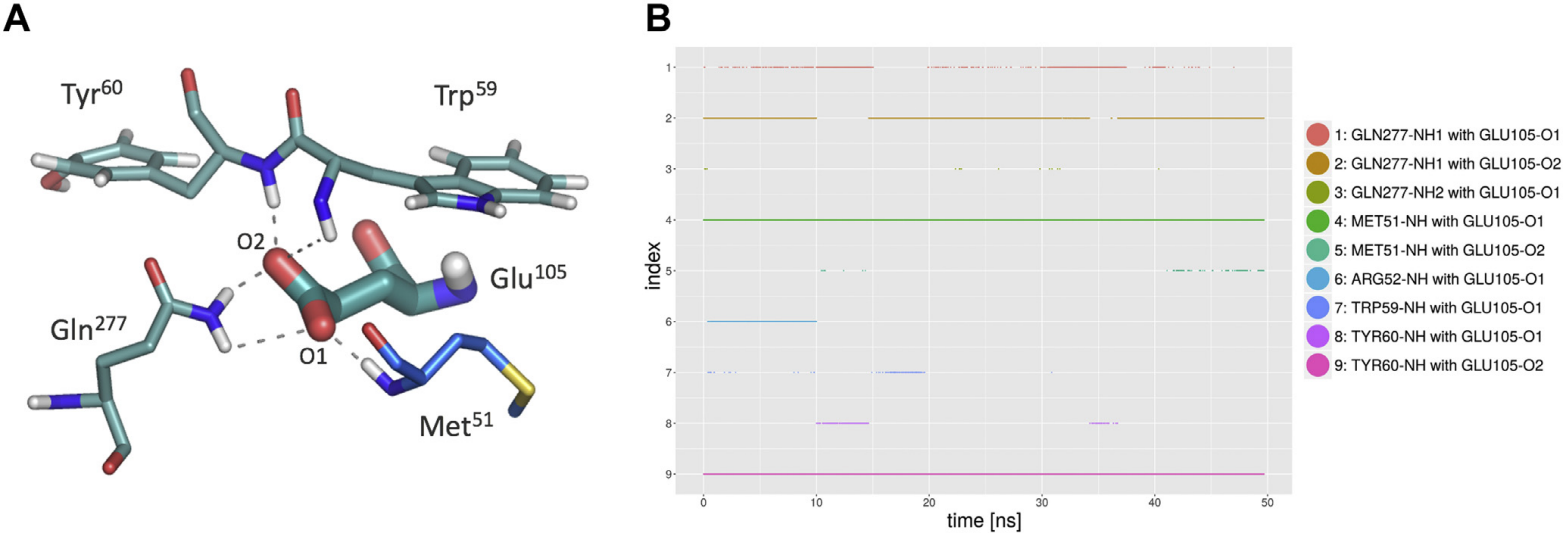
**Interactions of residue Glu**^**105**^**.***A*, Glu^105^ in a typical conformation, forming hydrogen bonds (through its two carboxylic oxygens O1 and O2) with the backbone NH-groups of Met^51^ (part of the *middle loop*, *blue*), Trp^59^ and Tyr^60^ and with the side chain amine group of Gln^277^. *B*, hydrogen bonding time series over a timeframe of 50 ns. The side chain of Glu^105^ is typically forming 4 to 6 hydrogen bonds at the same time. Total occurrences (id:%): 1:19.4, 2:77.0, 3:0.1, 4:96.3, 5:2.2, 6:18.5, 7:0.7, 8:3.1, 9:92.0.

The caspase has individual binding pockets for each residue on the unprimed site, except for P2. While there are no pockets for the prime site residues, they can crucially influence binding of the ligand and subsequent cleavage of the peptide bond by steric hinderance due to limited space at the active site. Especially the P1′ residue, which is in very close proximity to the catalytic cysteine (Cys^279^), has a crucial role. Small, branched residues in the P1′ site may hinder interactions between the cysteine and the site of hydrolysis, while long, polar side chains may hinder the ligand binding process itself.

**Figure 6:**
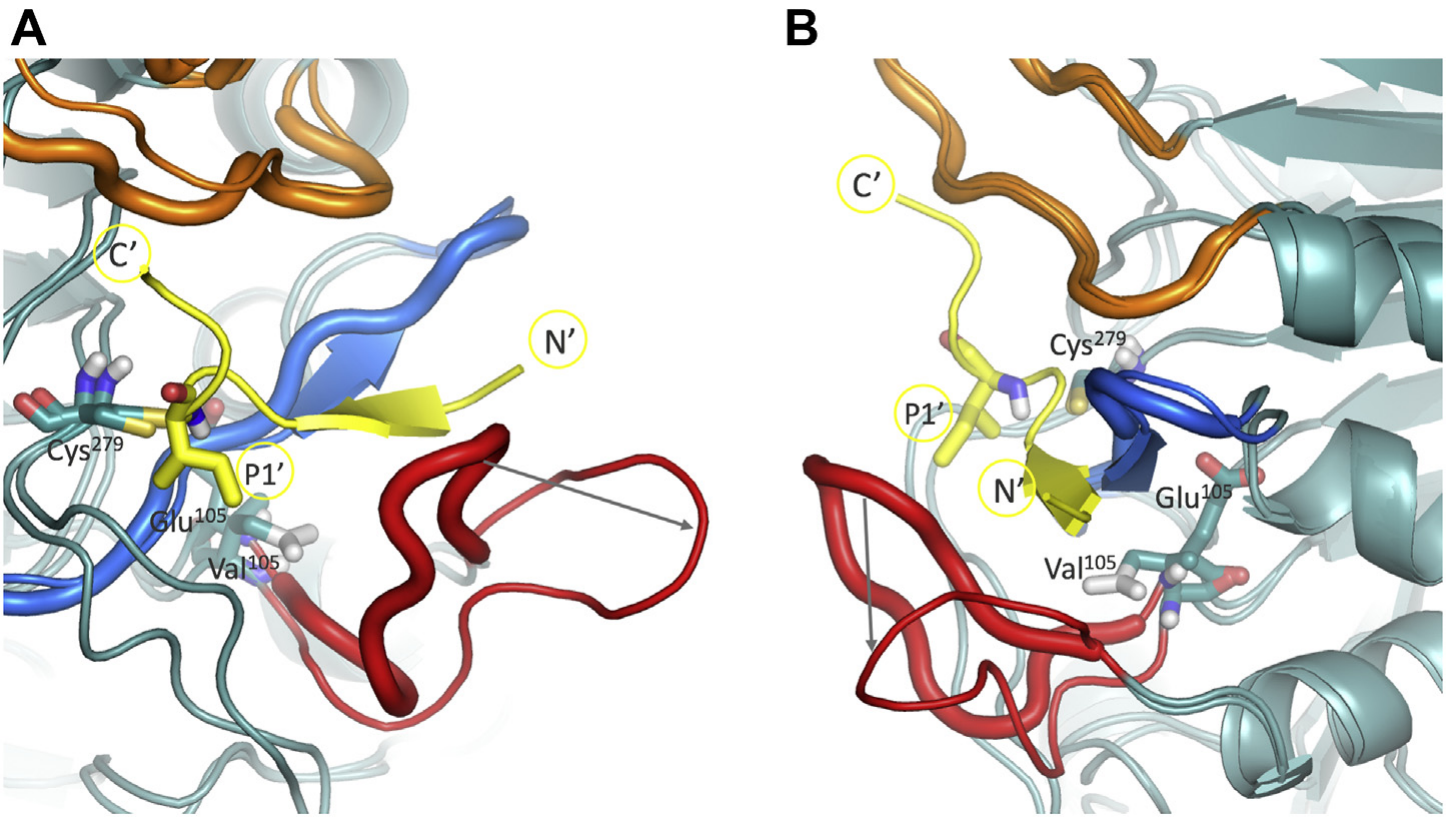
**Overlay of the structures of caspase-2 wild-type and S9 variant.** Ligand’s P1′ residue in close proximity to the catalytic cysteine (Cys^279^) displayed as *sticks*. *Thick loops* represent the wild-type caspase-2 structures (Glu^105^), while *thin loops* correspond to the S9 variant (Val^105^ mutation). Panel *A* and *B* show different representative conformations from the simulations. In both structure overlays, the distance between the ligand (*yellow*) and the *lower loop* (*red*) increases in the S9 variant. The loop reorientation is indicated with *gray arrows*.

The MD simulations showed that residue Glu^105^ (cp caspase-2 internal numbering) has an important stabilizing effect in the outer hydrophobic core of the caspase. It is positioned at the lower loop, as shown in Figure 4*B*. The lower loop from Gly^93^ to Glu^105^ in cp caspase-2 interacts with the unprimed side of the ligand. The glutamate residue accepts hydrogen bonds from several donors, mainly Met^51^, Tyr^60^, and Gln^277^, as shown in Figure 5*A*, which stabilize the lower loop in the binding pocket. The time series of hydrogen bonds with Glu^105^ as hydrogen bond acceptor from a 50 ns MD simulation is shown in Figure 5*B*.

In the S9 variant, the valine with its aliphatic, nonpolar side chain, in contrast to the negatively charged glutamate, does not act as a hydrogen bond acceptor. The mutation leads to a reorientation of the lower loop, which is visualized in Figure 6. The distance between the loop and the ligand increased, causing less tight binding of the substrate. The binding pocket is more flexible as the lower loop is destabilized. Furthermore, the active site is more solvated, which increases interactions of the ligand with the solvent. Overall, this allows the enzyme to be more promiscuous toward the P1′ residue at the catalytic step.

### Selection system design with P1′ variation

To improve the tolerance of a protease for a specific P1′ residue, a selection system in which this residue can be modified is necessary. Several approaches were pursued to change the original starting methionine as P1′ residue of cp ATCase but all resulted in loss of the enzyme’s activity. The issue was not only to find out which mutations cp ATCase tolerates but also to take the influence of *E. coli* methionine amino peptidase (MAP) into account.

It was reported that the starting methionine of cp ATCase c227 is processed to about 50% in the untagged protein (29), suggesting that removal of this residue might be necessary for its activity. Our experiments corroborated that removal of the starting methionine of cp ATCase was a crucial prerequisite for its activity (experimental details can be found in the Supporting information). The severe structural constraints in this domain are incompatible with the original N-terminal methionine and only its removal by MAP allows correct folding of cp ATCase. Obviously, during our first selection, this methionine was removed after cleavage by cp caspase-2 rendering cp ATCase folding competent.

Selection for P1′ tolerance demands the deletion of the original starting Met^227^ (as every other amino acid cannot be removed by MAP and confers steric folding stress) and requires variation of the subsequent amino acid Thr^228^. It represents the actual N-terminus compatible with correct folding after removal of the starting methionine. Most amino acids are tolerated as substitute for Thr^228^, only a few bulky residues, namely phenylalanine, tyrosine, tryptophan, histidine, and lysine, seem to be incompatible with the formation of active cp ATCase and can therefore not be used for a P1′-targeted selection.

### SARS-CoV-2 main protease M^pro^

In addition to the autoprotease N^pro^ and cp caspase-2, we tested PROFICS with the SARS-CoV-2 main protease M^pro^ (also 3C-like proteinase, 3CL^pro^). Its preferred recognition site (AVLQ-S, P4P3P2P1-P1′) (8) and a 6H tag were fused to the N-terminus of the cp ATCase c-chain to block the carbamoyltransferase’s activity. The main protease of SARS-CoV-2 was chosen because it is a highly interesting target for drug development (8), and we wanted to show that PROFICS can be used as a platform to identify drug-resistance-conferring mutations. Inhibitors that enter the cell and specifically block the proteolytic activity of M^pro^ prevent bacterial growth in the selection strain. Growth selection of a protease library in the presence of the inhibitor can detect mutations that cause resistance, as only cells with a mutated and drug resistant protease can reactivate cp ATCase and survive the selection. A limitation is that the system can only be used for substances that are nontoxic for the host (unpublished data).

Cells coexpressing M^pro^ and cp ATCase grew under selective conditions in the agar plate as well as in the shaking flask assay (Table 2). Untagged cp ATCase constructs with the original threonine (cp M-T228-ATCase) and with a serine mutation (cp M-T228S-ATCase) were used as positive controls. Tagged 6H-AVLQ-S-cp ATCase was expressed without protease as negative control.

**Table 2:** List of plasmids used for double transformations into *E. colipyr*− cells (pACYC vector with/without protease, pETDuet vector with ATCase) and ability to overcome pyrimidine auxotrophy

Substrate plasmid MCSI/MCSII of pETDuet-1	Protease plasmid	
	Empty pACYC (Mock)	pACYC M^pro^
pyrI/cp pyrB	a	a
pyrI/cp pyrB T228S	a	n. d.
pyrI/6H-AVLQ-S-cp pyrB	b	a

a Cells able to form distinct colonies on selective agar within 24 to 48 h.

b Cells not able to form colonies on selective agar within 24 to 48 h, n. d. not determined.

No cell growth was observed when the cells expressed only 6H-AVLQ-S-cp ATCase. Whereas cells expressing one of the untagged cp ATCase variants and cells coexpressing M^pro^ and 6H-AVLQ-S-cp ATCase grew under selective conditions and formed colonies after 24 to 48 h (Fig. S3).

## Discussion

PROFICS is a bacterial selection system for proteases, which depends on the reactivation of an essential enzyme by proteolytic tag cleavage to overcome auxotrophy.

The system uses auxotroph, *pyrBI* deficient *E. coli* cells containing a cp ATCase inactivated by N-terminal fusion of an autoprotease or a short tag including a protease recognition site. The tagged cp ATCase is inactive but specific cleavage by a protease restores its activity. The growth of the cells is dependent on the activity and specificity of the protease and allows the selection of active variants with altered properties.

The intrinsic counter selection of the *in vivo* selection ensures that only variants with low off-target activity are found. A protease with relaxed specificity would cleave crucial *E. coli* proteins and reduce or prevent cell growth. Several prerequisites have to be tested before selections: The overexpression of the protease must not have negative or toxic effects on the host cell. On the other hand, the protease has to be stable and active under the conditions during selection in living cells. It is also important that the recognition site of the protease is not cleaved by host cell enzymes, as otherwise cp ATCase activity could be restored without efficient expression of the transformed protease.

Proteases that fulfill these criteria can be “PROFICSed” either on agar plates or in liquid cultures from large mutant libraries in a simple overnight process. Bacterial growth is a very simple readout, which does not require detection of fluorescence or colorimetric measurements. In liquid culture improved variants can outgrow others, as shown in our example wherein a single caspase mutant was enriched. This simplifies the selection but also bears the risk that additional variants may be overlooked. Noncompetitive plate assays can overcome this issue.

The size and quality of the mutant library as well as the transformation efficiency of the selection host are crucial points for the establishment of a bacterial selection system. They define how many variants can be analyzed per experiment and thereby the chance to find an improved variant. We generated a customized system with competent cells that already contain one expression vector to avoid inefficient double transformations. The cloning and transformation procedures together with our selection assays create a system for the establishment of large libraries and the selection of variants with an easy setup, little hands-on time, and a fast readout.

PROFICS enables the directed evolution of proteases by repeating the steps of mutation and selection. The high transformation efficiency and the fast growth of *E. coli* allow alteration of the characteristics of a protease in just a few days.

By modifying the N-terminus of cp ATCase, we created the possibility for directed evolution of a protease and used it to improve the qualities of a cp caspase-2 designed for an industrial platform production process (9). A platform process ought to be applicable to any target, independent of the N-terminus of the protein of interest. Our selection toolbox comprises protease substrates with all but five P1′ residues. These all have large, bulky side chains and probably hinder the folding or function of cp ATCase.

**Figure 7:**
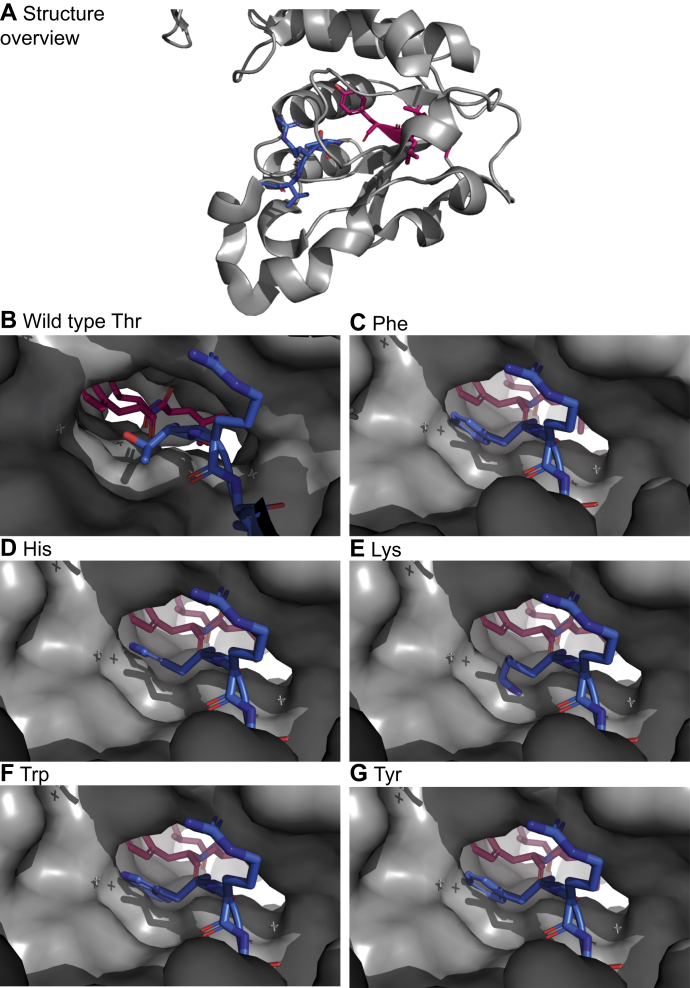
**Visualization of the structure of cp ATCase and the location of the new termini (N-terminus *blue*, C-terminus *red*).** The starting methionine was deleted and the subsequent residue exchanged to various amino acids. *A*, overview of the structure; *B*–*G*, position of the termini inside a core structure; *B*, N-terminus with (wild-type) threonine residue; *C*–*G*, N-terminal threonine was changed to large, bulky residues that block the “tunnel” and possibly change the protein structure.

The termini of the circularly permuted enzyme are located inside a β-sheet and our fusion experiments have proven that proper folding is easily obstructed as the available space and flexibility are limited. Figure 7 shows the termini inside the structure. The beta strand is surrounded by a “tunnel” formed by helices. Large, bulky residues do not fit well into this tunnel and possibly prevent correct folding by changing the structure of the ATCase.

However, if P1′ selections with one of the five incompatible amino acids are required, our mutation and selection strategy can be applied to cp ATCase variants with those respective residues at their N-termini, to find active mutants that tolerate these bulky residues.

We found that processing of the starting methionine by *E. coli* methionine aminopeptidase seems to be necessary for ATCase activity. A single additional amino acid at the N-terminus of the catalytic subunit of cp ATCase hinders its activity, suggesting very strict space limitations. This enables the application of the system for the selection of optimized exopeptidases, such as amino- or carboxypeptidases. Aminopeptidases could be selected for altered P1′ tolerance or increased activity. For the latter, a cp ATCase construct with multiple methionine residues at its N-terminus could be used.

The selection pressure of PROFICS can be adapted by changing the P1′ residue of the substrate, depending on the required preferences of a protease.

The potency of PROFICS was demonstrated by the selection of an improved, highly efficient caspase variant. After just one round of mutation and selection, we saw a large increase in enzymatic activity (*k*_*cat*_/*K*_*M*_ values) and promiscuity toward the P1′ residue used in the selection. These changes gave the variant an advantage over other mutants in the library and enabled faster growth of the host cells.

By conducting an unbiased mutation procedure without using structural knowledge or rational design to predict and identify ideal regions for mutation, our bacterial selection approach yielded a variant with a single, relatively unusual amino acid exchange that was found to confer strikingly improved catalytic properties (about fivefold *k*_*cat*_ increase with standard substrate Abz-VDVAD↓GA-Dap(Dnp)) and P1′ tolerance (up to 25-fold *k*_*cat*_ increase). *In silico* analyses suggest that the influence of the mutation results from destabilizing effects on a substrate recognition loop and more flexible binding of the substrate. The random mutation exchanged a charged glutamic acid residue to a hydrophobic, branched-chain valine. The two amino acids have very different properties and would not likely be selected in rational design based on the protein structure, unless all amino acids are considered (38). The impact of such harsh mutations, which are in general supposed to have unfavorable consequences, is usually difficult to predict based only on structural models. This mutation is also rather unlikely to occur in nature without negative impact on the protein’s function as seen for the sickle cell anemia Glu to Val mutation in the hemoglobin beta chain (39). Glutamates are usually exchanged to aspartic acids or polar residues such as glutamine in neutral mutations, as these residues have more similar properties to the original amino acid (40).

The two caspase-2 variants originating from *in silico* studies (26) both showed increased cleavage activities, mainly for substrates with branched and nonpolar P1′ residues. Interestingly, the mutation found with PROFICS without using structural data is located in a completely different region of the enzyme. Although the P1′ tolerance of all three variants is improved, the effect is larger in the variant found with PROFICS. It might be beneficial to combine the mutations found with the different methods and to analyze if their effects are additive. A combination of *in silico* and random approach might discover further improved variants.

The concept of PROFICS was validated with three entirely different proteases, proving that, with small adaptions, it is applicable for the selection of various proteases with *cis* and *trans* cleavage activity. Its potency was demonstrated by finding a variant with significantly increased activity and changed specificity. Furthermore, soluble production optimization for proteases, by improvement of expression tags or even codon usage, is also possible by choosing selection criteria that require particularly high levels of soluble, active protease, for example, by providing unfavorable P1′ residues such as proline in the cp ATCase reporter enzyme.

## Experimental procedures

Composition of buffers and media and sequences of all constructs and primers can be found in the Supporting information. All chemicals were obtained from ROTH (Carl Roth GmbH + Co KG), primers were ordered at Sigma Aldrich (Merck KGaA). For site-specific mutations, amino acid deletions and insertions, the NEB Q5 Site-Directed Mutagenesis Kit (New England BioLabs (NEB)) was used. Correct cloning was verified by DNA sequencing (Eurofins Genomics).

### *E. coli* strains

*E. coli* NovaBlue competent cells (Novagen, Merck KGaA) were used for cloning of all constructs. For the selection system, an ATCase-deficient strain was generated (*E. coli pyr*−). The ATCase operon was knocked out in *E. coli* BL21(DE3) cells (Novagen) using the Quick&Easy *E. coli* Gene Deletion Kit (Gene Bridges GmbH) and exchanged to a kanamycin resistance cassette. Electro competent *E. coli pyr*− cells containing a plasmid coding for cp ATCase with respective protease cleavage site were prepared to avoid double transformations (36, 41).

### Construction of cp ATCase plasmids

The coding sequence of *E. coli* ATCase regulatory subunit *pyrI* (UniprotKB ID P0A7F3) was amplified by PCR from BL21(DE3) genomic DNA using primers pyrI_genome_forw and pyrI_genome_rev. The gene was cloned into the multiple cloning site I (MCSI) of the bacterial expression vector pETDuet-1 (Novagen) with restriction sites *Nco*I and *Not*I. A possible caspase cleavage site (DQVD) in the PyrI protein was eliminated by exchanging Asp^73^ to glutamic acid.

For coexpression of the two subunits, the cDNA of *cp pyrB c227* (based on wild-type sequence UniprotKB ID P0A786, as described by (29)) was synthesized (biomers.net GmbH) and cloned with restriction sites *Nde*I and *Xho*I into the MCSII of the pETDuet-1 vector containing the regulatory subunit.

### N^pro^-ATCase constructs

The coding sequence of the N-terminal protease of the polyprotein of *Classical swine fever virus (strain Alfort) (CSFV) (Hog cholera virus)* (UniProtKB - P19712) was inserted N-terminal of the cp c-chain of the cp ATCase pETDuet construct.

### Caspase and M^pro^ ATCase constructs

A 6H tag, followed by a short GSG linker for flexibility, and a caspase-2 recognition site (VDVAD) were inserted at the N-terminus of the cp c-chain by site-directed mutagenesis. A second construct was created with the caspase-14 recognition site (WEHD).

For M^pro^ cleavages, the VDVAD recognition site was mutated to AVLQ, Met^227^ deleted and Thr^228^ exchanged to serine with site-directed mutagenesis using primers AVLQS_forw and AVLQS_rev.

### Construction of caspase selection plasmid

The cp caspase-2 construct previously described (9, 26) was cloned into the MCSII of a pACYCDuet-1 vector (Novagen) with restriction sites *Nde*I and *Xho*I.

### Construction of M^pro^ plasmid

The coding DNA of SARS-CoV-2 3C-like proteinase (also main protease M^pro^; part of replicase polyprotein 1ab UniProtKB – P0DTD1) was codon optimized for *E. coli* with the GeneArt online tool, synthesized (Thermo Fisher Scientific) and cloned into the MCSII of a pACYCDuet-1 vector with restriction sites *Nde*I and *Xho*I.

### Preparation of caspase mutant libraries

#### Error-prone PCR

The error-prone PCR (ep PCR) protocol was adapted from Wilson and Keefe (42). The PCR reaction contained 5 mM MgCl_2_, 0.2 mM MnCl_2_, 1 mM dCTP and dTTP, 0.2 mM dGTP and dATP, 0.5 μM of each primer (ep_caspase_forw and ep_caspase_rev), 5 pg/μl template caspase DNA, 1× Colorless GoTaq reaction buffer, and 0.0125 U/μl GoTaqG2 polymerase (Promega). The conditions for thermo cycling were: 95 °C for 2 min, followed by 25 cycles of 95 °C for 30 s, 47 °C for 30 s, and 72 °C for 2 min, and a final step at 72 °C for 5 min. PCR product was purified using the QIAquick PCR Purification Kit and a MiniElute column (both Qiagen (QIAGEN N. V.)), the concentration was determined using a spectrophotometer (NanoDrop ND-1000, Thermo Fisher Scientific).

#### Overlap extension PCR

The mutated caspase gene was cloned into a pACYCDuet-1 vector with overlap extension PCR (oe PCR) following the protocol of Kim *et al.* (43).

The vector was multiplied with Q5 Hot Start High-Fidelity 2X Master Mix (NEB) using primers vector_forw and vector_rev with an annealing temperature of 51 °C. The PCR product was purified using QIAquick spin columns (Qiagen) and the concentration determined as described above.

For oe PCR purified vector and ep PCR products were mixed at a molecular ratio of 1:1 (44.9 ng/μl vector, 10 ng/μl caspase) and amplified with Q5 Hot Start High-Fidelity 2X Master Mix and primers ep_caspase_forw and vector_rev (0.5 μM each). The first step was at 98 °C for 2 min, followed by 15 cycles of 98 °C for 20 s, 51 °C for 20 s, and 72 °C for 2.5 min, and a final step at 72 °C for 5 min. The product of the oe PCR was purified by gel extraction and DNA concentration was determined with a spectrophotometer.

#### Ligation

Purified linear oe PCR product (4 ng/μl) was ligated with T4 DNA ligase (Promega) at 16 °C overnight. The ligation product was purified using QIAquick PCR Purification Kit and QIAquick spin columns (both Qiagen).

### Selection procedure—shaking flask and agar plate assay

The purified construct or library (1 μl) was transformed by electroporation into a 40 μl aliquot of freshly prepared *E. coli pyr*− cells. For experiments with caspase and M^pro^, the competent cells contained the cp ATCase plasmid with the respective cleavage site. All media, except SOC, were supplemented with kanamycin and ampicillin. For the experiments with caspase and M^pro^, chloramphenicol was added to maintain the protease plasmids.

After recovery in SOC medium for 60 min, the cells were carefully washed and resuspended in 500 μl PBS buffer to remove the rich medium containing pyrimidines. To determine the size and quality of the gene library and the number of mutations per construct, 1 μl of the cell suspension was plated on TY agar plates and after incubation, several colonies were analyzed by sequencing.

For the flask assay, the cell suspension was diluted in supplemented M9 medium in a baffled Erlenmeyer shaking flask and incubated shaking at 30 °C until turbidity was clearly visible (24–48 h). Flask assays were used to enrich mutants with improved growth. To obtain single colonies for sequence analysis, dilutions of the liquid culture were plated on supplemented M9 agar plates, incubated at 30 °C, and single colonies analyzed by sequencing.

For the agar plate assay, the washed cell suspension was appropriately diluted, plated on supplemented M9 agar plates, and incubated at 30 °C for 24 to 48 h.

IPTG concentrations in liquid culture and in agar plates between 0.025 and 1 mM were tested. Expression of proteins was confirmed by SDS-PAGE analysis (data shown in Supporting information).

### Expression and enzyme activity measurements

The DNA of the selected variant was extracted and purified. The caspase was expressed and purified with IMAC methods as described previously (26). Kinetics were measured with a peptide substrate based FRET assay as reported (9). In brief, A FRET peptide substrate with the sequence Abz-VDVADXA-Dap(Dnp) was used (Bachem AG), where Abz is the fluorophore 2-Aminobenzoyl, VDVAD is the caspase-2 recognition site, X is the P1′ site where all 20 proteinogenic amino acids were used, A is alanine as P2′, Dap is diamino-propionic acid, and Dnp is 2,4-dinitrophenyl as the quencher. The assay was performed in 50 mM HEPES, 150 mM NaCl, pH 7.2 at 25 °C, with varying substrate concentrations of 10, 20, 50, 100, and 200 μM and a constant enzyme concentration of 1 μM purified cp caspase-2. From the initial slope of fluorescence (Tecan Infinite M200 Pro plate reader), the product generation was calculated and TableCurve 2D v5 was used to fit the data to the Michaelis–Menten kinetic equation.

### Molecular dynamics simulations

MD simulations were performed using the GROMOS11 molecular simulation software (44). A covalent bond was added between the catalytic cysteine and the substrate peptide, effectively simulating the tetrahedral intermediate state of the catalytic process. In terms of steady-state kinetics, this means that the effects of mutations on *k*_*cat*_, rather than *K*_*M*_ should be captured. Proteins and ligands were described using the GROMOS 54a8 parameter set (45), water was treated explicitly and described by means of the simple point charge (SPC) model (46). Simulations were carried out using periodic boundary conditions (PBC) based on rectangular simulation boxes. Equations of motions were integrated using the leap-frog algorithm (47), the SHAKE algorithm (48) was applied for bond length constraints allowing for integration time-steps of 2 fs. Nonbonded interactions were treated using a twin range cutoff (short range 0.8 nm, long-range 1.4 nm). For long-range interactions, a reaction field contribution (49) with a relative dielectric permittivity of 61, as appropriate for the SPC water model, was added (50). Initial velocities were sampled from a Maxwell–Boltzmann distribution at 60 K. The system was slowly heated up to 298.15 K in five discrete steps of 100 ps each and finally further equilibrated for 2 ns at 298.15 K. In the subsequent production simulation, the temperature was maintained at 298.15 K by weak coupling to two external heat baths for the solute and the solvent separately. The production simulation of the wild-type caspase-2 was performed for 50 ns. The S9 mutant was created from the equilibrated structure by slowly changing the glutamate to valine at position 105 alchemically (changing the Hamiltonian gradually). A production simulation for the mutated structure was subsequently performed as well.

### Analyses of MD trajectories

Hydrogen bonding analysis was performed using the gromos++ program (51). A maximum distance of 0.25 nm between the hydrogen and the acceptor and a minimum donor–hydrogen–acceptor angle of 135° were chosen as acceptance criteria.

Structural visualizations of the coordinate trajectories were performed in PyMOL (52).

## Data availability

All data described are presented either within the article or in the Supporting Information.

## Supporting information

This article contains supporting information (53).

## Conflict of interest

The authors declare that they have no conflicts of interest with the contents of this article.
