# Supplementary material for: PROFICS: A bacterial selection system for directed evolution of proteases
Source: J Biol Chem. 2021 Aug 19;297(4):101095. doi: 10.1016/j.jbc.2021.101095 (PMC8446807; doi:10.1016/j.jbc.2021.101095)
Supplement: Supplemental Figures S1–S3 and Tables S1, S3–S5 [file mmc1.pdf]

# PROFICS: A bacterial selection system for directed evolution of proteases

Christina Kröß[1,2,§], Petra Engele[1,2,§], Bernhard Sprenger[1,2], Andreas Fischer[1,3], Nico Lingg[1,3], Magdalena Baier[2], Christoph Öhlknecht[1,4], Bettina Lier[1,4], Chris Oostenbrink[1,4], Monika Cserjan-Puschmann[1,3], Gerald Striedner[1,3], Alois Jungbauer[1,3], Rainer Schneider[1,2]

## List of material included

Additional experimental procedures for detection of protein expression in bacterial cells with N$^{pro}$, cp caspase-2 and M$^{pro}$

Figure S1 Expression control of cells containing cp ATCase and N$^{pro}$ fusion proteins

Figure S2 Expression control of cells containing cp ATCase fusion variants and cp caspase-2

Figure S3 Expression control of cells containing cp ATCase fusion variants and M$^{pro}$

Table S1 Selected cp caspase-2 mutants

Table S2 FRET assay results for cp caspase-2 S9 variant compared to cp caspase-2 (9) and *in silico* variant cp caspase-2 D323T H226A (25). Data provided in separate .xlsx format

Supporting materials and methods for P1' ATCase selections

Table S3 Activity of cp ATCase with different N-terminal amino acids

Table S4 Sequences of used oligonucleotides for PCR

Nucleotide and amino acid sequences of used constructs

Table S5 Buffer and media compositions

## Expression controls and cleavage of proteases in PROFICS selection system

### Western blot (N$^{pro}$-cp ATCase)

The proteins were separated by SDS-PAGE (NuPAGE 4-12 % Bis-Tris gel, Invitrogen, Thermo Fisher Scientific) and blotted onto a methanol activated PVDF membrane applying 30 V constant for 1 h. Blocking was executed in TBS-T (20 mM Tris Base, 150 mM NaCl, 0.05 % Tween, pH 7.6) with 5 % milk powder for one hour. Incubation with primary antibody (1:10,000 dilution of α-N$^{pro}$ antibody, produced in our laboratory, in blocking solution) was at 4 °C overnight. After washing with TBS-T, the membrane was incubated with secondary antibody (α-Goat IgG, Santa Cruz Biotechnology, Inc.; Dallas, TX, USA), diluted 1:4,000 in blocking solution, for 1 h. For detection SuperSignal® West Pico Luminol/Enhancer Solution and SuperSignal® West Pico Stable Peroxide Solution (Thermo Fisher Scientific) and the digital imaging system FUSION FX7 from Peqlab (VWR International; Radnor, PA, USA) were used.

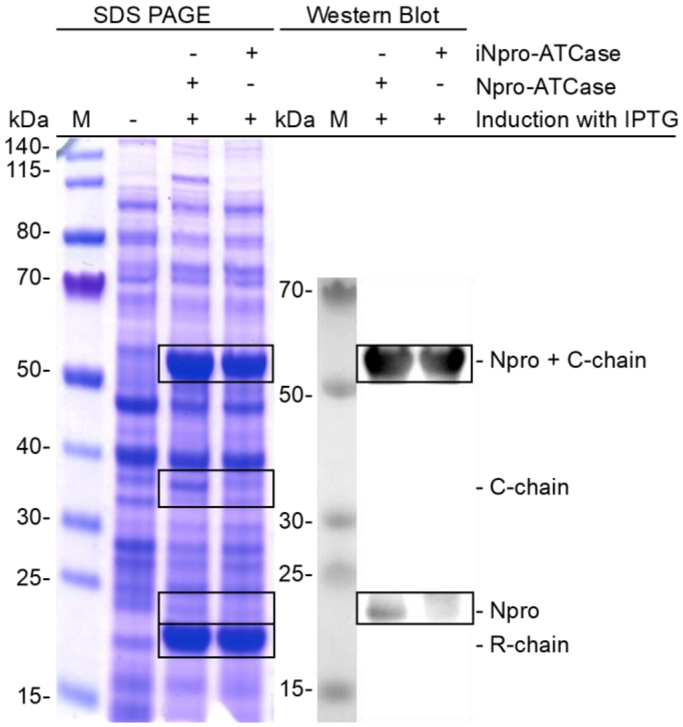


Figure S1: SDS-PAGE and Western blot with antibody against N$^{pro}$ of *E. coli pyr-* cells containing a fusion of active N$^{pro}$ and cp ATCase (Npro-ATCase) or a fusion of inactive N$^{pro}$ and cp ATCase (iNpro-ATCase). Samples were taken before and after induction of expression. Fusion proteins and the ATCase regulatory chain (R-chain) are expressed in both cell types. Only in the cells with active N$^{pro}$ the fusion is cleaved and the ATCase catalytic chain (C-chain) and N$^{pro}$ were detected separately.

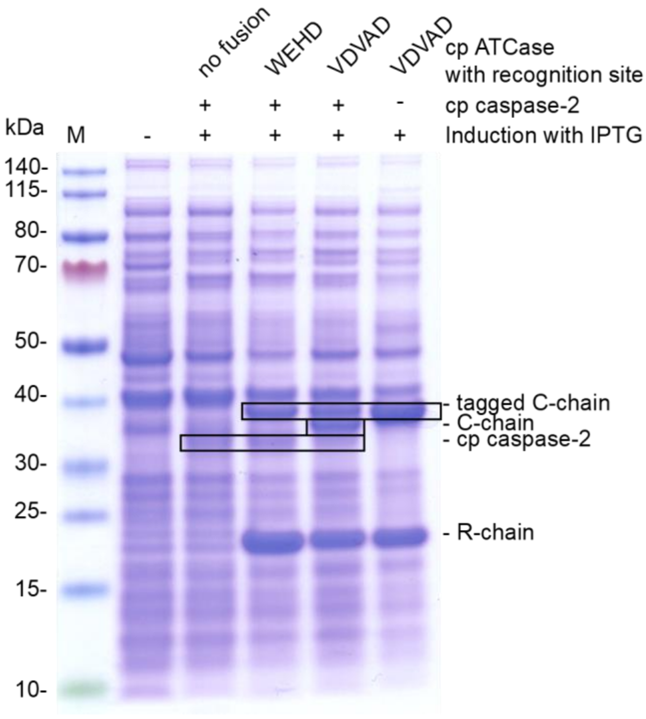


Figure S2: SDS-PAGE of *E. coli pyr-* cells containing cp ATCase with different recognition sites (WEHD and VDVAD) in their fusion tags; cp ATCase without fusion and cp caspase-2 without cp ATCase were used as controls. Samples were taken before and after induction of expression. The regulatory chain is expressed in all ATCase containing cells. In addition, the cleaved cp c-chain is visible in the cells containing cp caspase-2 together with 6H-VDVAD-cp ATCase. The uncleaved (tagged) cp c-chain is visible in the cells without cp caspase-2 and in the cells with the WEHD recognition sequence.

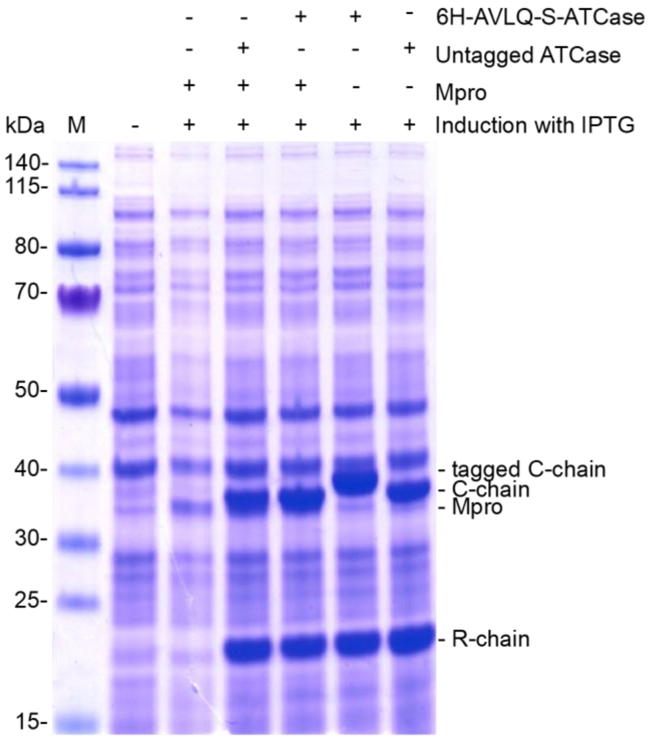


Figure S3: SDS-PAGE of *E. coli pyr-* cells containing cp ATCase with a fusion tag comprising the AVLQ-S recognition site. Cells expressing cp ATCase without fusion and only $M^{pro}$ are shown as controls. Samples were taken before and after induction of expression. The regulatory chain is expressed in all four samples containing a cp ATCase variant. The cleaved and uncleaved catalytic chain can be seen in the cells containing 6H-AVLQ-S-cp ATCase with or without $M^{pro}$ respectively.

## Selected variants from caspase mutation

On selective plates colonies with varying sizes were obtained. Colonies larger in size, which were suspected to contain a caspase variant conferring an advantage over others because of the cells' faster growth were analyzed in more detail.

Table S1: Mutations of variants selected from cp caspase-2 library. The library was transformed in *E. coli pyr-* cells containing tagged cp ATCase. Total volume of the transformation was 500 µl. Aliquots of 250 and 25 µl were pipetted into flasks with selective media (approach Fa and Fb), aliquots of 200 (Pa), and 20 µl (Pb) were plated on selective agar plates. 1 µl was plated on non-selective TY-agar and colonies sequenced to estimate the size of the library and the number of mutations.

| | No. | Mutations | Silent mutations |
|---|---|---|---|
| **Colonies from non-selective conditions** | I | none | 0 |
| | II | C44R T145A V238F | 0 |
| | III | K89 F100S H116Q L194Q F217L | 0 |
| **Agar plate assay Pa** | 1 | L45Q K136R | 3 |
| | 2 | E105V | 1 |
| | 3 | none | 0 |
| | 4 | none | 0 |
| | 5 | T126S | 0 |
| | 6 | R35S Q144R | 2 |
| **Agar plate assay Pb** | 13 | K26R | 3 |
| | 14 | none | 2 |
| | 15 | none | 1 |
| | 16 | none | 2 |

| | | | |
|---|---|---|---|
| **Flask assay Fa** | 7 | E105V | 4 |
| | 9 | E105V | 1 |
| | 10 | E105V | 1 |
| **Flask assay Fb** | 8 | F147L | 0 |
| | 11 | L149R V201A | 0 |
| | 12 | none | 1 |

## FRET Results

Table S2: Michaelis Menten kinetics measured using FRET substrate VDVADXA at 1 µM enzyme concentration. (n.d. not determined). cp caspase-2 S9 variant compared to cp caspase-2 (data from (9)) and *in silico* variant cp caspase-2 D323T H226A (data from (25))

Data provided in separate .xlsx file

## Additional experiments for P1' selections

### Construction of cp ATCase plasmids

For the cp MA-ATCase construct an alanine was inserted at the N-terminus of cp *pyrB* by site directed mutagenesis using primers MA-pyrB_forw and MA-pyrB_rev.
The original $Thr^{228}$ was mutated to all 19 canonical amino acids with site directed mutagenesis using degenerate primers M-X-ATCase_forw and M-X-ATCase_rev, resulting in cp M-(T→X)-ATCase constructs.

For the deletion variant the $Met^{227}$ in the cp *pyrB* was removed with site directed mutagenesis.
A gene library of $N^{pro}$-(M→X)-cp ATCase constructs was cloned with degenerate primers Npro-(M-X)-ATCase_forw and Npro-(M-X)-ATCase_rev.

Methionine deletion variants (6H-VDVAD-ΔM-X-cp ATCase) were created with site directed mutagenesis.

### Experiments to establish P1' toolbox

For the P1' toolbox experiments untagged cp ATCase constructs (cp MA-ATCase, cp M-(T→X)-ATCase), were transformed into *E. coli pyr-* cells without protease). Cell growth under selective conditions confirmed ATCase activity.

First an alanine was added to the N-terminus of cp ATCase (cp MA-ATCase) to see if the desired P1' amino acid could simply be inserted upstream of the original terminus. The activity of this construct was tested with the agar plate assay, no colony formation was observed.
Next, we generated and selected a gene library in which we mutated the second residue $Thr^{228}$ to all canonical amino acids (cp M-(T→X)-ATCase). Active cp ATCase variants with nine different amino acids in the mutated position were found (A, C, G, P, Q, R, S, T, V). Nearly all these residues are readily accepted by MAP (54).
To express cp ATCase variants without starting methionine we generated a gene library of $N^{pro}$-cp ATCase. The fusion enabled us to exchange the starting residue of the cp ATCase ($N^{pro}$-(M→X)-cp ATCase). This approach was unsuccessful, only variants starting with methionine survived the selection.

These experiments indicated that the activity of the cp ATCase depends on removal of the starting methionine rather than on the variation of the $Thr^{228}$ residue (Table S3). Apparently, the spatial

restrictions of the structure and the charges introduced with the new termini do not tolerate much variation. We concluded that removal of the methionine could increase the flexibility of the protein chain. To test the activity of different P1' cp ATCase variants without their native N-terminal methionine the fusion protein with caspase cleavage tag was used, as it has another start codon upstream of the tag. The disadvantage is, that the process is not only influenced by proper cp ATCase folding but also by the P1' tolerance of the caspase. The methionine deletion variants were generated with the ten P1' residues not found in the cp M-(T→X)-ATCase selection. Co-expression with cp caspase-2 under selective conditions confirmed the activity of 5 VDVAD-ΔM-X-cp ATCase variants (X = D, E, I, L, and N). No cell growth was observed on selective plates when the P1' was F, H, K, W, or Y after 48–72 hours of incubation.

To verify the results, a methionine deletion variant of $N^{pro}$-cp ATCase as well as the VDVAD-ΔM-cp ATCase together with cp caspase-2 were tested in the shaking flask assay. Cell growth was drastically increased in comparison to cells expressing the respective proteins without the deletion (data not shown). This confirms that the initial methionine is not necessary for enzymatic function but possibly even detrimental and that the subsequent amino acid of cp ATCase ($Thr^{228}$) can be mutated to nearly all canonical amino acids (Table S3), which allows the generation of a toolbox for selection of proteases with a specific P1' tolerance.

Table S3: Activity of cp ATCase, X is any amino acid despite methionine.

| **N-terminal amino acids of cp c-chain** | **Cell growth** |
|---|---|
| MTRVQKERL… | Yes (original construct) |
| $N^{pro}$-MTRVQKERL… | Yes |
| $N^{pro}$-XTRVQKERL… | No, except when X=M |
| $N^{pro}$-TRVQKERL… | Yes, improved compared to original construct |
| MA-MTRVQKERL… | No |
| MXRVQKERL… | Yes, when X accepted by MAP |
| $MH_6$GSGVDVAD-MTRVQKERL… | Yes, when co-expressed with cp caspase-2 |
| $MH_6$GSGVDVAD-TRVQKERL… | Yes, when co-expressed with cp caspase-2 improved compared to $MH_6$GSGVDVAD-MTRVQKERL… |
| $MH_6$GSGVDVAD-XRVQKERL… | Yes, when co-expressed with cp caspase-2 except X is F, H, K, W, Y |

## Sequences

### Primer Sequences

Table S4: Oligonucleotides used for cloning experiments

| **Primer name** | **Sequence 5'→3'** |
|---|---|
| pyrI_genome_forw | TATACCATGGGCACACACGATAATAAATTGCAG |
| pyrI_genome_rev | TATAGCGGCCGCTTAATTGGCCAGCACCAC |
| MA-pyrB_forw | atggccATGACCCGCGTGCAAAAA |
| MA-pyrB_rev | ATGTATATCTCCTTCTTATACTTAACTAATATAC |
| M-X-ATCase_forw | TATACATATGnnnCGCGTGCAAAAAGAG |
| M-X-ATCase_rev | TCTCCTTCTTATACTTAACTAATATAC |
| Npro-(M-X)-ATCase_forw | CACTAGTTGCnhnACCCGCGTGC |
| Npro-(M-X)-ATCase_rev | ACCCACAGTGGACAGTTAG |
| AVLQS_forw | gcagagcCGCGTGCAAAAAGAGCGT |

| | |
|---|---|
| AVLQS_rev | agaacggcGCCGCTGCCATGATGATG |
| ep_caspase_forw (5' phosphorylated) | AGATATACATATGCACCA |
| ep_caspase_rev | gcatccgatATCTTATTA |
| vector_forw (5' phosphorylated) | CCTTCTTATACTTAACTAATAT |
| vector_rev | TAATAAGATatcggatgc |

**Construct sequences**

**Active $N^{pro}$-cp ATCase**

**Nucleotide sequence: *pyrI***

ATGGGCACAC ACGATAATAA ATTGCAGGTT GAAGCTATTA AACGCGGCAC GGTAATTGAC 60
CATATCCCCG CCCAGATCGG TTTTAAGCTG TTGAGTCTGT TCAAGCTGAC CGAAACGGAT 120
CAGCGCATCA CCATCGGTCT GAACTTACCT TCTGGCGAGA TGGGCCGCAA AGATCTGATC 180
AAAATCGAAA ATACCTTTTT GAGTGAAGAC CAGGTAGATC AACTGGCATT GTATGCGCCG 240
CAAGCCACGG TTAACCGTAT CGACAACTAT GAAGTGGTGG GTAAATCGCG CCCAAGTCTG 300
CCGGAGCGCA TCGACAATGT GCTGGTCTGC CCGAACAGCA ACTGTATCAG CCATGCCGAA 360
CCGGTTTCAT CCAGCTTTGC CGTGCGAAAA CGCGCCAATG ACATCGCGCT CAAATGCAAA 420
TACTGTGAAA AAGAGTTTTC CCATAATGTG GTGCTGGCCA ATTAA 465

**Protein sequence: PyrI**

MGTHDNKLQV EAIKRGTVID HIPAQIGFKL LSLFKLTETD QRITIGLNLP 50
SGEMGRKDLI KIENTFLSED QV**D**QLALYAP QATVNRIDNY EVVGKSRPSL 100
PERIDNVLVC PNSNCISHAE PVSSSFAVRK RANDIALKCK YCEKEFSHNV 150
VLAN 154

**Nucleotide sequence: $N^{pro}$-*pyrB***

ATGGAACTCA ATCATTTCGA ACTGCTCTAC AAAACTAGCA AGCAAAAACC TGTTGGCGTT 60
GAAGAGCCGG TCTACGATAC TGCAGGTCGT CCTCTTTTTG GGAATCCGTC CGAAGTGCAC 120
CCCCAGTCAA CCCTCAAGCT TCCCCATGAC CGCGGACGCG GTGACATTCG TACAACGCTG 180
CGCGATCTGC CTCGTAAAGG CGATTGTCGC TCTGGAAACC ACCTAGGTCC GGTGTCGGGC 240
ATTTACATTA AACCAGGTCC CGTCTATTAC CAAGACTACA CTGGTCCGGT TTACCATCGT 300
GCACCTCTGG AATTCTTTGA TGAAGCTCAA TTTTGCGAAG TGACTAAACG TATTGGCCGT 360
GTAACCGGTT CGGACGGGAA ACTGTACCAC ATCTACGTGT GCGTTGATGG CTGTATCCTG 420
CTGAAACTCG CGAAGCGCGG AACCCCTCGC ACCCTGAAAT GGATCCGTAA CTTCACTAAC 480
TGTCCACTGT GGGTCACTAG TTGCATGACC CGCGTGCAAA AAGAGCGTCT GGACCCGTCC 540
GAGTACGCCA ACGTGAAAGC GCAGTTTGTT CTTCGCGCCA GCGATCTCCA CAACGCCAAA 600
GCCAATATGA AAGTGCTGCA TCCGCTGCCG CGTGTTGATG AGATTGCGAC GGATGTTGAT 660
AAAACGCCAC ACGCCTGGTA CTTCCAGCAG GCAGGCAACG GGATTTTCGC TCGCCAGGCG 720
TTACTGGCAC TGGTTCTGAA TCGCGCTAAT CCGCTATATC AGAAACATAT CATTTCCATA 840
AACGACCTTA GTCGCGATGA CCTTAATCTG GTGCTGGCGA CAGCGGCGAA ACTGAAAGCA 900
AACCCGCAAC CAGAGCTGTT GAAGCACAAA GTCATTGCCA GCTGTTTCTT CGAAGCCTCT 960
ACCCGTACCC GCCTCTCTTT CGAAACTTCC ATGCACCGCC TGGGTGCCAG CGTGGTGGGC 1020
TTTTCCGACA GCGCCAATAC ATCACTGGGT AAAAAGGGCG AAACGCTGGC CGATACCATT 1080
TCGGTTATCA GCACTTACGT TGATGCGATA GTGATGCGTC ATCCGCAGGA AGGTGCGGCG 1140
CGCCTGGCCA CCGAGTTTTC CGGCAATGTA CCGGTACTGA ATGCCGGTGA TGGCTCCAAC 1200
CAACATCCGA CGCAAACCTT GCTGGACTTA TTCACTATTC AGGAAACCCA GGGGCGTCTG 1260
GACAATCTCC ACGTCGCAAT GGTTGGTGAC CTGAAATATG GCCGCACCGT TCACTCCCTG 1320
ACTCAGGCGT TAGCGAAGTT CGACGGCAAC CGTTTTTACT TCATCGCGCC GGACGCGCTG 1380
GCAATGCCGC AATACATTCT GGATATGCTT GATGAAAAAG GGATCGCCTG GAGTCTGCAC 1440
AGCTCTATTG AAGAAGTGAT GGCGGAAGTT GACATCCTGT ACTAATAA 1488

**Protein Sequence: $N^{pro}$-pyrB**

MELNHFELLY KTSKQKPVGV EEPVYDTAGR PLFGNPSEVH PQSTLKLPHD 50
RGRGDIRTTL RDLPRKGDCR SGNHLGPVSG IYIKPGPVYY QDYTGPVYHR 100
APLEFFDEAQ FCEVTKRIGR VTGSDGKLYH IYVCVDGCIL LKLAKRGTPR 150
TLKWIRNFTN CPLWVTSC**M**T RVQKERLDPS EYANVKAQFV LRASDLHNAK 200
ANMKVLHPLP RVDEIATDVD KTPHAWYFQQ AGNGIFARQA LLALVLNRAN 250

PLYQKHIISI NDLSRDDLNL VLATAAKLKA NPQPELLKHK VIASCFFEAS 300
TRTRLSFETS MHRLGASVVG FSDSANTSLG KKGETLADTI SVISTYVDAI 350
VMRHPQEGAA RLATEFSGNV PVLNAGDGSN QHPTQTLLDL FTIQETQGRL 400
DNLHVAMVGD LKYGRTVHSL TQALAKFDGN RFYFIAPDAL AMPQYILDML 450
DEKGIAWSLH SSIEEVMAEV DILY 474

**Inacitve N$^{pro}$-cp ATCase**
**Protein sequence: iN$^{pro}$-pyrB**
MELNHFELLY KTSKQKPVGV EEPVYDTAGR PLFGNPSEVH PQSTLKLPHD 50
RGRGDIRTTL RDLPRKGDCR SGNHLGPVSG IYIKPGPVYY QDYTGPVYHR 100
APLEFFDEAQ FCEVTKRIGR VTGSDGKLYH IYVCVDGCIL LKLAKRGTPR 150
TLKWIRNFTN CPLAVTSC**M**T RVQKERLDPS EYANVKAQFV LRASDLHNAK 200
ANMKVLHPLP RVDEIATDVD KTPHAWYFQQ AGNGIFARQA LLALVLNRAN 250
PLYQKHIISI NDLSRDDLNL VLATAAKLKA NPQPELLKHK VIASCFFEAS 300
TRTRLSFETS MHRLGASVVG FSDSANTSLG KKGETLADTI SVISTYVDAI 350
VMRHPQEGAA RLATEFSGNV PVLNAGDGSN QHPTQTLLDL FTIQETQGRL 400
DNLHVAMVGD LKYGRTVHSL TQALAKFDGN RFYFIAPDAL AMPQYILDML 450

**cp ATCase c227 PyrI D73E**
**MCS I Protein Sequence:**
MGTHDNKLQV EAIKRGTVID HIPAQIGFKL LSLFKLTETD QRITIGLNLP 50
SGEMGRKDLI KIENTFLSED QV**E**QLALYAP QATVNRIDNY EVVGKSRPSL 100
PERIDNVLVC PNSNCISHAE PVSSSFAVRK RANDIALKCK YCEKEFSHNV 150
VLAN 154

**MCS II Protein Sequence:**
**M**TRVQKERLD PSEYANVKAQ FVLRASDLHN AKANMKVLHP LPRVDEIATD 50
VDKTPHAWYF QQAGNGIFAR QALLALVLNR ANPLYQKHII SINDLSRDDL 100
NLVLATAAKL KANPQPELLK HKVIASCFFE ASTRTRLSFE TSMHRLGASV 150
VGFSDSANTS LGKKGETLAD TISVISTYVD AIVMRHPQEG AARLATEFSG 200
NVPVLNAGDG SNQHPTQTLL DLFTIQETQG RLDNLHVAMV GDLKYGRTVH 250
SLTQALAKFD GNRFYFIAPD ALAMPQYILD MLDEKGIAWS LHSSIEEVMA 300
EVDILY 306

**cp caspase-2**
**Nucleotide Sequence:**
ATGCACCATC ATCACCATCA TGGCAAAAAT CATGCAGGTA GTCCGGGTTG TGAAGAAAGC 60
GCAGCAGGTA AAGAAAAACT GCCGAAAATG CGTCTGCCGA CCCGTAGCGA TATGATTTGT 120
GGTTATGCAT GTCTGAAAGG CACCGCAGCA ATGCGTAATA CCAAACGTGG TAGCTGGTAT 180
ATTGAAGCAC TGGCACAGGT TTTTAGCGAA CGTGCATGTG ATATGCATGT TGCAGATATG 240
CTGGTTAAAG TGAACGCCCT GATTAAAGAT CGTGAAGGTT ATGCACCGGG TACAGAATTT 300
CATCGTTGTA AAGAAATGAG CGAGTATTGT AGCACCCTGT GTCGTCATCT GTACCTGTTT 360
CCGGGTCATC CTCCGACCGG ATCCGGTCCG GTTTGTCTGC AGGTTAAACC GTGTACACCG 420
GAATTTTATC AGACCCATTT TCAGCTGGCA TATCGTCTGC AGAGCCGTCC GCGTGGTCTG 480
GCACTGGTTC TGAGCAATGT TCATTTTACC GGTGAAAAAG AACTGGAATT TCGTAGCGGT 540
GGTGATGTTG ATCATAGTAC CCTGGTTACC CTGTTTAAAC TGCTGGGTTA TGACGTTCAT 600
GTTCTGTGTG ATCAGACCGC ACAAGAAATG CAAGAGAAAC TGCAGAATTT TGCACAGCTG 660
CCTGCACATC GTGTTACCGA TAGCTGTATT GTTGCACTGC TGAGCCATGG TGTTGAAGGT 720
GCAATTTATG GTGTGGATGG CAAACTGCTG CAACTGCAAG AAGTGTTTCA GCTGTTTGAT 780
AATGCAAATT GTCCGAGCCT GCAGAATAAA CCGAAAATGT TTTTTATCCA GGCCTGCCGT 840
GGTGATGAAA CCGATCGTGG TGTTGATCAG CAGGATTAAT AA 882

**Protein sequence:**
MHHHHHHGKN HAGSPGCEES AAGKEKLPKM RLPTRSDMIC GYACLKGTAA 50
MRNTKRGSWY IEALAQVFSE RACDMHVADM LVKVNALIKD REGYAPGTEF 100
HRCKEMSEYC STLCRHLYLF PGHPPTGSGP VCLQVKPCTP EFYQTHFQLA 150
YRLQSRPRGL ALVLSNVHFT GEKELEFRSG GDVDHSTLVT LFKLLGYDVH 200
VLCDQTAQEM QEKLQNFAQL PAHRVTDSCI VALLSHGVEG AIYGVDGKLL 250
QLQEVFQLFD NANCPSLQNK PKMFFIQACR GDETDRGVDQ QD 292

**S9 (cp caspase-2 E105V)**

**Nucleotide sequence:**

```
ATGCACCATC ATCACCATCA TGGCAAAAAT CATGCAGGTA GTCCGGGTTG TGAAGAAAGC  60
GCAGCAGGTA AAGAAAAACT GCCGAAAATG CGTCTGCCGA CCCGTAGCGA TATGATTTGT 120
GGTTATGCAT GTCTGAAAGG CACCGCAGCA ATGCGTAATA CCAAACGTGG TAGCTGGTAT 180
ATTGAAGCAC TGGCACAGGT TTTTAGCGAA CGTGCATGTG ATATGCATGT TGCAGATATG 240
CTGGTTAAAG TGAACGCCCT GATTAAAGAT CGTGAAGGTT ATGCACCGGG TACAGAATTT 300
CATCGTTGTA AAGTAATGAG CGAGTATTGT AGCACCCTGT GTCGTCATCT ATACCTGTTT 360
CCGGGTCATC CTCCGACCGG ATCCGGTCCG GTTTGTCTGC AGGTTAAACC GTGTACACCG 420
GAATTTTATC AGACCCATTT TCAGCTGGCA TATCGTCTGC AGAGCCGTCC GCGTGGTCTG 480
GCACTGGTTC TGAGCAATGT TCATTTTACC GGTGAAAAAG AACTGGAATT TCGTAGCGGT 540
GGTGATGTTG ATCATAGTAC CCTGGTTACC CTGTTTAAAC TGCTGGGTTA TGACGTTCAT 600
GTTCTGTGTG ATCAGACCGC ACAAGAAATG CAAGAGAAAC TGCAGAATTT TGCACAGCTG 660
CCTGCACATC GTGTTACCGA TAGCTGTATT GTTGCACTGC TGAGCCATGG TGTTGAAGGT 720
GCAATTTATG GTGTGGATGG CAAACTGCTG CAACTGCAAG AAGTGTTTCA GCTGTTTGAT 780
AATGCAAATT GTCCGAGCCT GCAGAATAAA CCGAAAATGT TTTTTATCCA GGCCTGCCGT 840
GGTGATGAAA CCGATCGTGG TGTTGATCAG CAGGATTAAT AA 882
```

**Protein sequence:**

```
MHHHHHHGKN HAGSPGCEES AAGKEKLPKM RLPTRSDMIC GYACLKGTAA  50
MRNTKRGSWY IEALAQVFSE RACDMHVADM LVKVNALIKD REGYAPGTEF 100
HRCKVMSEYC STLCRHLYLF PGHPPTGSGP VCLQVKPCTP EFYQTHFQLA 150
YRLQSRPRGL ALVLSNVHFT GEKELEFRSG GDVDHSTLVT LFKLLGYDVH 200
VLCDQTAQEM QEKLQNFAQL PAHRVTDSCI VALLSHGVEG AIYGVDGKLL 250
QLQEVFQLFD NANCPSLQNK PKMFFIQACR GDETDRGVDQ QD 292
```

**SARS-CoV-2 M$^{pro}$**

**Nucleotide sequence**

```
ATGAGCGGTTTTCGTAAAATGGCATTTCCGAGCGGTAAAGTTGAAGGTTGTATGGTTCAGGTTACCTG
TGGCACCACCACACTGAATGGTCTGTGGCTGGATGATGTTGTTTATTGTCCGCGTCATGTTATTTGTA
CCAGCGAAGATATGCTGAACCCGAATTATGAAGATCTGCTGATTCGCAAAAGCAACCATAATTTTCTG
GTTCAGGCAGGTAATGTTCAGCTGCGTGTTATTGGTCATAGCATGCAGAATTGTGTGCTGAAACTGAA
AGTTGATACCGCCAATCCGAAAACGCCGAAATATAAGTTTGTTCGTATTCAGCCTGGTCAGACCTTTA
GCGTTCTGGCATGTTATAATGGTAGCCCGAGCGGTGTTTATCAGTGTGCAATGCGTCCGAATTTTACC
ATTAAAGGCAGCTTTCTGAATGGTAGCTGTGGTAGCGTTGGTTTCAACATTGATTATGATTGCGTGAG
CTTCTGCTATATGCATCACATGGAACTGCCGACCGGTGTTCATGCAGGCACCGATCTGGAAGGTAACT
TTTATGGTCCGTTTGTTGATCGTCAGACCGCACAGGCAGCAGGTACAGATACCACCATTACCGTTAAT
GTTCTGGCCTGGCTGTATGCAGCAGTTATTAATGGTGATCGCTGGTTTCTGAATCGTTTTACAACAAC
CCTGAACGATTTTAATCTGGTGGCCATGAAATATAACTATGAACCGCTGACACAGGATCATGTTGATA
TTCTGGGTCCGCTGAGCGCACAGACCGGTATTGCAGTTCTGGATATGTGTGCAAGCCTGAAAGAACTG
TTACAGAATGGTATGAATGGTCGTACAATTCTGGGTAGCGCACTGCTGGAAGATGAATTCACCCCGTT
TGATGTTGTGCGTCAGTGTAGCGGTGTTACCTTTCAGTAATAA
```

**Protein sequence**

```
MSGFRKMAFPSGKVEGCMVQVTCGTTTLNGLWLDDVVYCPRHVICTSEDMLNPNYEDLLIRKSNHNFL
VQAGNVQLRVIGHSMQNCVLKLKVDTANPKTPKYKFVRIQPGQTFSVLACYNGSPSGVYQCAMRPNFT
IKGSFLNGSCGSVGFNIDYDCVSFCYMHHMELPTGVHAGTDLEGNFYGPFVDRQTAQAAGTDTTITVN
VLAWLYAAVINGDRWFLNRFTTTLNDFNLVAMKYNYEPLTQDHVDILGPLSAQTGIAVLDMCASLKEL
LQNGMNGRTILGSALLEDEFTPFDVVRQCSGVTFQ
```

Table S5 Composition of used buffers and media

| Buffer/Media | Composition |
| --- | --- |
| **PBS** (Phosphate buffered saline, pH 7.4) | 137 mM NaCl, 2.7 mM KCl, 10 mM $Na_2HPO_4$, 1.8 mM $KH_2PO_4$ |
| **TY** (Tryptone yeast) medium | 1 % (w/v) peptone, 0.7 % (w/v) yeast extract, 0.25 % (w/v) NaCl |
| **SOC** (Super optimal broth with catabolite repression) | 20 g/L tryptone, 5 g/L yeast extract, 10 mM NaCl, 2.5 mM KCl, 10 mM $MgCl_2$, 20 mM glucose |
| **Supplemented M9 minimal medium** | 50 mM $Na_2HPO_4$, 20 mM $KH_2PO_4$, 10 mM NaCl, 1 mM $MgSO_4$, 0.1 mM $CaCl_2$, 0.4 % (w/v) glucose, 0.5 % (w/v) casamino acids, 20 mM $NH_4Cl$, 10 µg/ml $FeSO_4$, vitamins (0.001 mg/ml of each biotin, thiamine, riboflavin, pyridoxine, niacinamide), 0.025–1 mM IPTG |
| Used antibiotics | Kanamycin (50 µg/ml)<br>Ampicillin (100 µg/ml)<br>Chloramphenicol (34 µg/ml) |
